# Different Residues on the Surface of the *Methanothermobacter thermautotrophicus* MCM Helicase Interact with Single- and Double-Stranded DNA

**DOI:** 10.1155/2010/505693

**Published:** 2010-12-01

**Authors:** Nozomi Sakakibara, Rajesh Kasiviswanathan, Zvi Kelman

**Affiliations:** ^1^Center for Advanced Research in Biotechnology, University of Maryland Biotechnology Institute, 9600 Gudelsky Drive, Rockville, MD 20850, USA; ^2^Laboratory of Viral Diseases, NIAID, NIH, 4 Center Drive, Bethesda, MD 20892, USA; ^3^Laboratory of Molecular Genetics, National Institute of Environmental Health Sciences, 111 TW Alexander Drive, Research Triangle Park, NC 27709, USA; ^4^Department of Cell Biology and Molecular Genetics, Institute for Bioscience and Biotechnology Research, University of Maryland, 9600 Gudelsky Drive, Rockville, MD 20850, USA

## Abstract

The minichromosome maintenance (MCM) complex is thought to function as the replicative helicase in archaea, separating the two strands of chromosomal DNA during replication. The catalytic activity resides within the C-terminal region of the MCM protein, while the N-terminal portion plays an important role in DNA binding and protein multimerization. An alignment of MCM homologues from several archaeal species revealed a number of conserved amino acids. Here several of the conserved residues located on the surface of the helicase have been mutated and their roles in MCM functions determined. It was found that some mutations result in increased affinity for ssDNA while the affinity for dsDNA is decreased. Other mutants exhibit the opposite effect. Thus, the data suggest that these conserved surface residues may participate in MCM-DNA interactions.

## 1. Introduction

**Figure 1:**
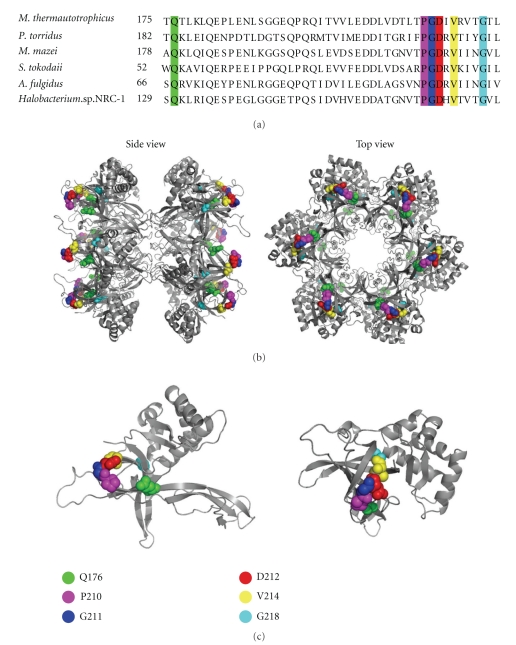
Locations of the amino acids mutated in the study. (a) An alignment of the amino acid sequences of the region of domain C analyzed in the study. The residues mutated are shown in color. Q176 (green), P210 (magenta), G211 (blue), D212 (red), V214 (yellow), G218 (cyan). The accession numbers used are: *Methanothermobacter thermautotrophicus*, NP_276876; *Picrophilus torridus*, YP_023995; *Methanosarcina mazei*, NP_633860; *Sulfolobus tokodaii*, NP_376352; *Archaeoglobus fulgidus*, NP_069353; *Halobacterium*, NP_280836. (b) Side and top views of the three-dimensional structure of the dodecameric N-terminal portion of the *M. thermautotrophicus* MCM protein (PDB ID: 1LTL [4]) are shown. The mutated residues are highlighted in colors as in (a). (c) Side and top views of the three-dimensional structure of the monomeric N-terminal portion of the *M. thermautotrophicus* MCM protein (PDB ID: 1LTL [4]) are shown. The mutated residues are highlighted in color as in (a).

The minichromosome maintenance (MCM) helicase is thought to function as the replicative helicase in eukarya and archaea. Most archaeal species contain a single MCM homologue with biochemical properties that are similar to the eukaryotic enzyme. Both archaeal and eukaryal MCM helicases exhibit ATP-dependent 3′-5′ helicase activity, can bind and translocate along single-stranded (ss) and double-stranded (ds) DNA, unwind DNA-RNA hybrids while translocating on the DNA strand, and can displace proteins from DNA (reviewed in [1–3]). The archaeal MCM protein can be divided into three parts; the N-terminal region, the AAA+ catalytic core, and a C-terminal region that may form a helix-turn-helix (HTH) domain [4–7]. The three-dimensional structure of the N-terminal portion of the *Methanothermobacter thermautotrophicus* and *Sulfolobus solfataricus* MCM proteins revealed a three domain architecture [4, 5]. Biochemical studies showed that domain A participates in regulating helicase activity, domain B participates in DNA binding, and domain C is involved in hexamer formation, DNA binding and communication between the N-terminal part and the catalytic domains (reviewed in: [1, 3]). Although the N-terminal portion of MCM is less conserved than the AAA+ region, an alignment of the N-terminal region from a number of archaeal species revealed the presence of several highly conserved residues, particularly in domain C (Figure 1(a)). Several of these residues have previously been reported to play an essential role in communicating between the N-terminal DNA binding and C-terminal catalytic activity [8, 9]. In this study, several conserved residues in domain C of the *M. thermautotrophicus* MCM protein were individually (except for one double mutant) mutated and the effects on MCM function were examined. All but one of the amino acids analyzed in the study are located on the surface of the helicase (Figure 1(b)), and thus they may play functional rather than structural roles. The amino acids analyzed in the study, highlighted in Figure 1(a), were Gln176, Pro210, Gly211, Asp212, Val214, and Gly218. It is shown here that while V214 or G218 to Ala substitutions do not significantly alter the MCM biochemical properties, P210 to Gly or G211 to Ala mutations promote enhanced binding to ssDNA and reduced binding to dsDNA. Q176 to Ala or D212 to Asn show better binding to dsDNA compared to ssDNA binding. The possible roles of these residues in MCM function are discussed. 

## 2. Materials and Methods

### 2.1. Materials

ATP and [*γ*-^32^P]ATP were obtained from GE Healthcare and oligonucleotides were synthesized by the NIST/UMD DNA synthesis facility.

### 2.2. Expression and Purification of MCM Mutant Proteins

All* M. thermautotrophicus* MCM mutant proteins used in this study are derivatives of the full-length enzyme and were generated using PCR-based site-directed mutagenesis as previously described [10]. All constructs contain a C-terminal His_6_-tag and were cloned into the NdeI and XhoI sites of the pET-21a vector (Novagen). The oligonucleotides used for the mutagenesis are shown in Supplementary Table 1. The wild-type and mutant proteins were overexpressed in *Escherichia coli* DE3 codon plus cells (Stratagene) at 37°C and purified on a Ni-column as previously described [11].

### 2.3. Gel Filtration

One hundred micrograms of wild-type or mutant MCM protein were fractionated on a superose-6 gel filtration column (HR10/30, GE Healthcare) pre-equilibrated with buffer containing 20 mM Tris-HCl (pH 7.5), 150 mM NaCl, and 10% glycerol. The column was run at 22°C with a flow rate of 0.2 ml/min. The presence of protein was determined by ultraviolet absorbance at 280 nm. 

### 2.4. ATPase Assay

ATPase activity was measured in reaction mixtures (15 *μ*l) containing 25 mM Hepes-NaOH (pH 7.5), 1 mM dithiothreitol, 5 mM MgCl_2_, 100 *μ*g/ml bovine serum albumin (BSA), and 1500 pmol of [*γ*-^32^P]ATP (3000 Ci/mmol; GE healthcare) and 10 or 30 nM MCM protein (as monomer) in the presence or absence of 50 ng ssDNA (5′-GCAGATAACAGTTGTCCTGGAGAACGACCTGGTTGACACCCTCACACCC-3′). After incubation at 60°C for 60 min, samples were placed on ice, then an aliquot (1 *μ*l) was spotted onto a polyethyleneimine cellulose thin layer plate, and ATP and *P*_*i*_ were separated by chromatography in 1 M formic acid and 0.5 M lithium chloride. The extent of ATP hydrolysis was quantitated by phosphorimager analysis. All ATPase assays were repeated three times, and their averages with standard deviations are shown in the figure.

### 2.5. Nitrocellulose Filter DNA Binding Assay

Single stranded and dsDNA substrates for filter binding assays were prepared by labeling the oligonucleotide using [*γ*-^32^P]ATP and T4 polynucleotide kinase. Unincorporated [*γ*-^32^P]ATP was removed from the DNA substrate by extraction from polyacrylamide gel as previously described [12]. For ssDNA binding a 49-mer oligonucleotide with the sequence 5′-GCAGATAACAGTTGTCCTGGAGAACGACCTGGTTGACACCCTCACACCC-3′ was used. For dsDNA the same oligonucleotide was hybridized to its complementary sequence. 

 Filter binding assays were carried out in a reaction mixture (20 *μ*l) containing 25 mM Hepes-NaOH (pH 7.5), 2 mM DTT, 10 mM MgCl_2_, 100 *μ*g/ml BSA, 50 fmol of  ^32^P-labeled ss or dsDNA substrates and 10, 30, or 90 nM of MCM protein (as monomer). After incubation at 60°C for 10 min the mixture was filtered through an alkaline-washed nitrocellulose filter (Millipore, HA 0.45 *μ*m) [13] which was subsequently washed with 20 mM Tris-HCl (pH 7.5). The radioactivity adsorbed to the filter was measured by liquid scintillation counting. All DNA binding experiments were repeated three times, and their averages with standard deviations are shown in the figure.

### 2.6. DNA Helicase Assay

Substrates for helicase assays were made as previously described [14] using the following oligonucleotides. For forked substrate the two oligonucleotides were 5′-GGGACGCGTCGGCCTGGCACGTCGGCCGCTGCGGCCAGGCACCCGATGGCGTTTGTTTGTTTGTTTGTTTGTTT-3′ and 5′-TTTGTTTGTTTGTTTGTTTGTTTGTTTGTTTGTTTGCCGACGTGCCAGGCCGACGCGTCCC-3′ and for the substrate with only a 3′-overhang region (flat substrate) the oligonucleotides used were 5′-CCGACGTGCCAGGCCGACGCGTCCC-3′ and 5′-GGGACGCGTCGGCCTGGCACGTCGGCCGCTGCGGCCAGGCACCCGATGGC-3′. 

 DNA helicase activity assays were measured in reaction mixtures (15 *μ*l) containing 20 mM Tris-HCl (pH 8.5), 2 mM DTT, 10 mM MgCl_2, _100 *μ*g/ml BSA, 3.33 mM ATP, 10 fmol (0.66 nM) of ^32^P-labeled substrate and 10, 30 or 90 nM MCM protein (as monomer) for flat substrate or 3, 9, or 27 nM MCM protein (as monomer) for forked substrate. Following incubation at 60°C for 1 hr the reactions were stopped by adding 5 *μ*l of loading buffer containing 1% SDS, 100 mM EDTA, 0.1% xylene cyanol, 0.1% bromophenol blue, and 50% glycerol and chilling on ice. Aliquots (10 *μ*l) were loaded onto an 8% native polyacrylamide gel in 0.5X TBE and electrophoresed for 40 min at 180 V. Gels were visualized and quantitated by phosphorimaging. All helicase experiments were repeated three times, and their averages with standard deviations are shown in the figure. 

### 2.7. Differential Scanning Calorimetry (DSC) Measurements

DSC was used to determine the denaturation temperature of the wild-type and mutant proteins as an indicator of the thermostability of the enzymes. The purified proteins were dialyzed at room temperature against buffer containing 20 mM potassium phosphate (pH 7.5), 100 mM NaCl and 10% glycerol. The protein concentrations were determined using an absorbance at 280 nm and a calculated extinction coefficient of A_280_ of 28730 cm^−1^·M^−1^. The solution outside the dialysis bag was retained and used as reference for the experiments. 

DSC measurements were performed using a VP-DSC Microcalorimeter from Microcal Inc. (Northampton, MA). The volumes of the solution and the reference vessel were 0.511 ml and the scan was either at slow rate (15 K hr^−1^) or medium rate (60 K hr^−1^), with the temperature ranging from 25–85°C. Since the scans of protein samples were irreversible, the second scan for each sample was used as the baseline. After subtraction of the baseline from the protein scan, the resulting raw data of differential powers as a function of time were divided by the scan rate to convert into a heat capacity as a function of temperature. Utilizing the EXAM program [15], a two state, A⇔B, transition model was then fitted to the heat capacity as a function of temperature scan to determine the van't Hoff enthalpy (Δ*H*_VH_) for the scan from the shape of the transition peak; a transition temperature (*T*_*m*_), and a calorimetric transition enthalpy (Δ*H*_cal_) was calculated from the area under the transition peak (mJ) divided by the moles of protein in the sample cell (concentration of protein × 0.511 ml). DSC scans on the samples were repeated twice.

## 3. Results

### 3.1. Mutations of the Conserved Residues Do Not Affect the Overall Structure of the MCM Proteins

**Figure 2:**
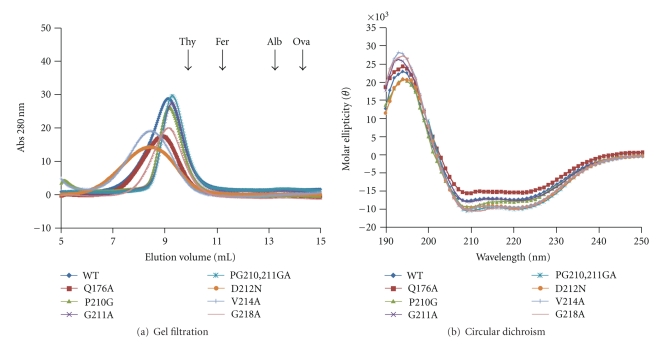
MCM mutant proteins retain their overall structural integrity. (a) Gel filtration analysis of wild-type and mutant proteins was performed as described in “Section 2”. The elution positions of size markers are shown: Thyroglobulin (Thy, 670,000 Da), ferritin (Fer, 440,000 Da), albumin (Alb, 67,000 Da) and ovalbumin (Ova, 45,000 Da). (b) Circular dichroism measurements were performed as described in “Section 2”.

In order to gain useful information from a mutant enzyme it is necessary to show that the mutation did not affect the overall structure of the molecule. All residues studied except G218 are located on the surface of the N-terminal part of MCM protein (Figure 1(b)) and thus are not expected to substantially alter the overall structure of the molecule. As shown in Figure 2 this indeed is the case. The size of all mutant proteins is similar to that of the wild-type enzyme (Figure 2(a)) although some mutant proteins elute earlier suggesting that they may have a greater tendency to form filaments. It was previously shown that the filaments are in equilibrium with the dodecameric and hexameric forms in a concentration-dependent manner [16]. The mutation may change the equilibrium between the different forms. According to the CD analysis, all mutant proteins retain similar overall secondary structure suggesting that the mutations introduced do not likely impact the overall structure of the proteins (Figure 2(b)). In addition, DSC analysis shows that all mutant proteins have similar thermal stability as the wild-type enzyme although the heat capacity change varies (Table 1). It is not clear what contributes to the large deviations and differences in the heat capacity change. Nevertheless, taken together, these results suggest that the mutations do not substantially alter the structures of the proteins.

### 3.2. The MCM Mutant Proteins Retain Helicase Activity

**Figure 3:**
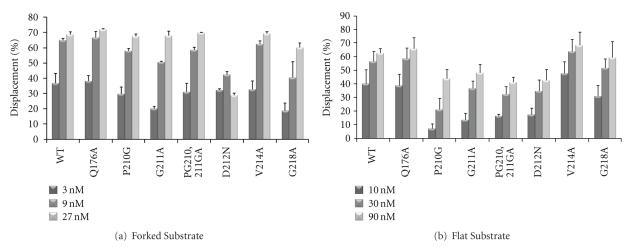
Helicase activity by the mutant MCM proteins. Helicase assays of wild-type and mutant MCM proteins were performed as described in “Section 2” with 10 fmol of forked (a) or flat (b) DNA substrates. In (a) the protein concentrations used were 3, 9 and 27 nM (as monomer) while in (b) they were 10, 30, and 90 nM (as monomer). The average results of three independent experiments with standard deviations are shown.

After establishing that the mutant proteins retain their overall structure, the effect of each mutation on MCM helicase activity was determined. As shown in Figure 3(a), most mutant proteins show wild-type enzyme-like activity on a forked DNA substrate. The exception is the D212N mutant enzyme, which decreases in activity as the concentration of the enzyme increases. It has been previously shown that MCM proteins are more active on forked substrate in comparison to substrate that contains only a 3′-overhang ssDNA region (flat substrate) (e.g., see: [11]). This difference in activity was previously used to analyze the effect of mild mutation on MCM helicase activity (e.g., [8]). Thus, helicase assays were also performed with flat substrate. As shown in Figure 3(b), the P210G, G211A, and PG210,211GA mutant enzymes have reduced helicase activity, particularly at low enzyme concentration. The D212N mutant protein also exhibits reduced helicase activity on a flat substrate but with no inhibition of helicase activity at high protein concentration (Figure 3(b)).

### 3.3. Mutations in the MCM Protein Affect DNA Binding

**Figure 4:**
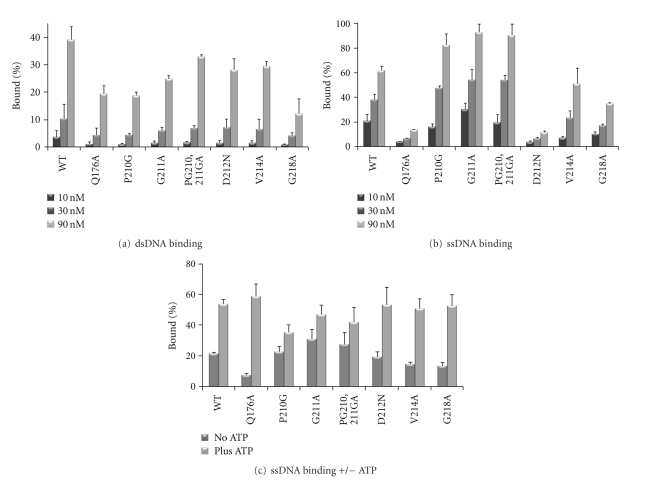
ss and dsDNA binding by the mutant MCM proteins. Nitrocellulose filter binding assays were performed as described under “Section 2” using 50 fmol of ^32^P-labled ds (a) or ss (b) DNA (49-mer) in the presence of 10, 30, and 90 nM of proteins (as monomer). (c) ssDNA binding assay contained 25 nM protein (as monomer) in the absence or presence of 1 mM ATP. The average results of three independent experiments with standard deviations are shown.

The data presented in Figure 3 show that several mutant enzymes have reduced helicase activity. This could be due to reduced DNA binding or reduced ATP hydrolysis as both are needed for DNA unwinding. To test this, DNA binding and ATPase assays were performed with the wild-type and mutant enzymes. All mutant proteins showed lower binding to dsDNA in comparison to the wild-type protein (Figure 4(a)). In particular, G218A showed ~25%, and Q176A, P210G, and G211A showed ~50% reduction compared to the duplex binding of the wild-type enzyme. When the ability of the mutant proteins to bind ssDNA it was found that the P210G, G211A or PG210,211GA mutant enzymes showed better binding than the wild-type protein while the D212N and Q176A exhibit reduced ability to bind ssDNA (Figure 4(b)). However, regardless their ability to bind DNA all proteins possess helicase activity (Figure 3), in particular the Q176A mutant enzyme which has helicase activity indistinguishable from the wild-type MCM on forked and flat substrate (Figure 3) but exhibits reduced ability to bind DNA (Figures 4(a) and 4(b)). It was previously shown that ATP enhances ssDNA binding by *M. thermautotrophicus* MCM [17, 18] and therefore the effect of ATP on ssDNA binding by the mutant enzymes were evaluated (Figure 4(c)). In the presence of ATP all proteins exhibit similar DNA binding activity. The ability of the mutant proteins to bind ssDNA in the presence of ATP may explain why some mutant proteins had wild-type helicase activity while no measurable ssDNA binding could be detected in the absence of ATP.

### 3.4. Diverse Effect of MCM Mutations on ATPase Activity

**Figure 5:**
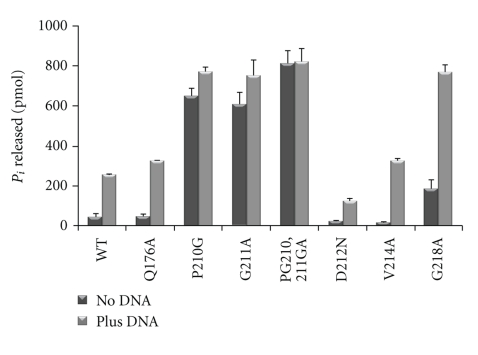
ATPase activity by the mutant MCM proteins. ATPase activities of wild-type and mutant MCM proteins were determined as described in “Section 2” using 30 nM of MCM (as monomer) in the presence or absence of 50 ng of ssDNA. The average results of three independent experiments with standard deviations are shown.

MCM proteins, including *M. thermautotrophicus* MCM, utilize ATP hydrolysis to translocate along DNA. The ATPase activity of *M. thermautotrophicus* MCM is stimulated in the presence of DNA [19–21]. The helicase and DNA binding experiments described above suggest that ATPase activity may be impaired in some of the mutants. As shown in Figure 5, the P210G, G211A, and PG210,211GA mutant proteins exhibit much greater (>10 fold) basal ATPase activity (in the absence of DNA) compared to the wild-type enzyme. Stimulation by DNA cannot be assessed for these proteins as maximal ATPase activity levels are presence in the absence of DNA. It is interesting to note, however, that those three mutant proteins (P210G, G211A, and PG210,211GA) also exhibit better ssDNA binding in comparison to the wild-type enzyme (Figure 4(b)).

### 3.5. The Cdc6 Protein Inhibits the Helicase Activity of MCM Mutant Enzymes

The residues studied could also play a role in protein-protein interactions. The archaeal Cdc6 proteins were shown to interact with MCM and to inhibit helicase activity [11, 22, 23]. The *M. thermautotrophicus* genome contains two Cdc6 homologues, Cdc6-1 and Cdc6-2, and both proteins were shown to inhibit MCM helicase activity [11]. It is possible that the surface residues mutated in the current study interact with the Cdc6 protein. To address this possibility the effect of Cdc6 proteins on the helicase activity by the mutant proteins was evaluated. As shown in supplementary Figure 1 Cdc6 proteins inhibit helicase activity of all mutant proteins to a similar extent suggesting that the mutated residues are not needed for the interaction with the Cdc6 protein.

## 4. Discussion

The role of several conserved residues in the *M. thermautotrophicus* MCM protein was examined using mutant enzymes. Mutations P210G, G211A and PG210,211GA exhibit elevated DNA binding (Figure 4) and ATPase activity (Figure 5). The P210 and G211 are located on the surface of domain C where they face the AAA+ catalytic domains in close proximity to helix 2 [3, 7]. Deletion of part of helix 2, known as the helix 2 insertion (H2i), resulted in an enzyme with no helicase activity and increased affinity for DNA [24]. It is possible that mutations of P210 and G211 affect the local structure of helix 2 and/or H2i. These local alterations are likely to be mild, as helicase activity could be detected with all three mutant enzymes (P210G, G211A, and PG210,211GA).

While the P210G, G211A, and PG210,211GA mutant enzymes demonstrated increased ssDNA binding they exhibit reduction in binding to dsDNA (Figure 4). The Q176A and D212N mutant proteins, on the other hand, exhibited the opposite effect, showing better binding to dsDNA than to ssDNA (Figure 4). The MCM protein may adopt a particular conformation when it binds ssDNA and a different one when it binds dsDNA. The P210G, G211A, and PG210,211GA mutations may cause the MCM proteins to adopt a conformation that favors binding to ssDNA while the Q176A and D212N may stabilize the other conformation. 

The reduction in ssDNA binding observed with the Q176A mutant protein could be restored in the presence of ATP (Figure 4(c)). Furthermore, in size exclusion chromatography, the Q176A mutant protein showed a relatively broad elution profile (Figure 2(a)), suggesting that the mutation may cause destabilization of the dodecameric ring. Q176 is located at the end of the *β*-sheet 7 (*β*7), which runs across domains B and C. Domain B was implicated in both DNA binding via the Zinc finger [10, 18] and dodecamer formation [16, 25]. It is possible that mutating Q176A may affect the structure and/or orientation of domain B in relation to the rest of the molecule thus affecting both DNA binding and the stability of the dodecamer.

 It is well established that the positively charged residues inside the central cavity of the hexameric MCM are responsible for both ss and dsDNA binding [4, 26]. It has also been reported that the helicase interacts with DNA via residues that are located on the surface of the MCM [27, 28]. Although it is not yet clear how, or where, the MCM protein binds to ss or dsDNA outside of the central cavity, the data presented here may suggest that the interactions, at least in part, are via P210, G211, and Q176. The data also suggest that this interaction on the surface of MCM may play a regulatory role in switching between ss and dsDNA binding. These interactions on the surface of the molecule, however, are not essential for helicase activity *in vitro*. In the future, when the methods are developed, it will be of interest to study the effect of these mutations *in vivo*.

## Supplementary Material

Supplementary Table 1 shows the oligonucleotides used to generate the mutant proteins using PCR-mediated mutagenesis.
Supplementary Figure 1 shows the effect of the two *M. thermautotrophicus* Cdc6 proteins on the helicase activity of the wild-type and mutant MCM proteins.

## Figures and Tables

**Table 1:** DSC analyses of wild-type and mutant proteins.

	*T*_*m*_ ave (°C)	Δ*H*_VH_ (kJ/mole)	Δ*H*_cal_ (kJ/mole)
Wild-type	66.2 ± 1.7*	1246.8 ± 159.0	530.6 ± 3.1
Q176A	66.2 ± 0.8	886.0 ± 25.2	248.2 ± 56.1
P210G	66.1 ± 0.2	1003.7 ± 89.5	242.7 ± 135.5
G211A	67.2 ± 0.2	1105.5 ± 21.9	335.5 ± 120.1
PG210,211GA	65.6 ± 1.1*	1137.5 ± 64.3	174.8 ± 60.3
D212N	68.1 ± 0.3	1191.4 ± 370.0	373.7 ± 156.5
V214A	65.7 ± 0.7	1066.0 ± 168.3	393.1 ± 142.2
G218A	65.9 ± 0.9	675.9 ± 136.4	425.8 ± 205.2

*Average of medium and slow scan.

## Abbreviations

CD: Circular dichroism,
DSC: Differential scanning calorimetry,
dsDNA: Double-stranded DNA,
MCM: Minichromosome maintenance,
ssDNA: Single-stranded DNA.
