# Supplementary material for: Different Residues on the Surface of the Methanothermobacter thermautotrophicus MCM Helicase Interact with Single- and Double-Stranded DNA
Source: Archaea. 2010 Dec 1;2010:505693. doi: 10.1155/2010/505693 (PMC2997501; doi:10.1155/2010/505693)
Supplement: Supplementary file 1 — Supplementary Table 1 shows the oligonucleotides used to generate the mutant proteins using PCR-mediated mutagenesis. Supplementary Figure 1 shows the effect of the two M. thermautotrophicus Cdc6 proteins on the helicase activity of the wild-type and mutant MCM proteins. [file 505693.f1.pdf]

Supplementary Table 1. List of primers used to generate mutants

Mutation	Sequence (5'-3')
Q176A F	TTC CTG GAC ACC GCG ACA CTG AAA CTC
Q176A R	GAG TTT CAG TGT CGC GGT GTC CAG GAA
P210G F	CTG GTT GAC ACC CTC ACA GGT GGG GAT ATT GTG AGG GTG ACC
P210G R	GGT CAC CCT CAC AAT ATC CCC ACC TGT GAG GGT GTC AAC CAG
G211A F	CTG GTT GAC ACC CTC ACA CCC GCT GAT ATT GTG AGG GTG ACC
G211A R	GGT CAC CCT CAC AAT ATC AGC GGG TGT GAG GGT GTC AAC CAG
PG210,211GA F	CTG GTT GAC ACC CTC ACA GGT GCT GAT ATT GTG AGG GTG ACC
PG210,211GA R	GGT CAC CCT CAC AAT ATC AGC ACC TGT GAG GGT GTC AAC CAG
D212A F	CTC ACA CCC GGG AAT ATT GTG AGG GTG ACC
D212A R	GGT CAC CCT CAC AAT ATT CCC GGG TGT GAG
V214A F	CCC GGG GAT ATT GCA AGG GTG ACC GGC
V214A R	GCC GGT CAC CCT TGC AAT ATC CCC GGG
G218A F	GTG AGG GTG ACC GCT ACC CTC AGG ACG
G218A R	CGT CCT GAG GGT AGC GGT CAC CCT CAC
