# Supplementary figures and images for: Different Residues on the Surface of the Methanothermobacter thermautotrophicus MCM Helicase Interact with Single- and Double-Stranded DNA
Source: Archaea. 2010 Dec 1;2010:505693. doi: 10.1155/2010/505693 (PMC2997501; doi:10.1155/2010/505693)

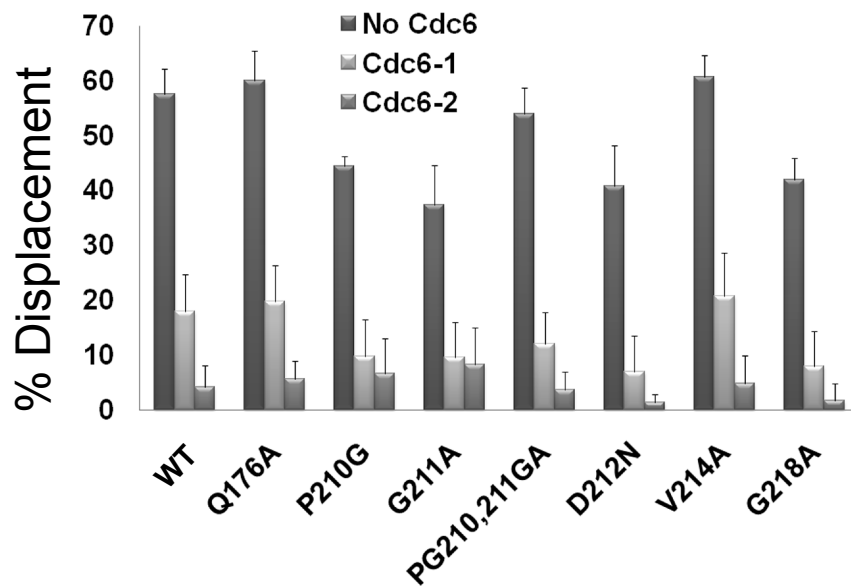

Supplementary Figure 1

Supplement: Supplementary file 2 [file 505693.f2.pdf]
